# Challenges in managing Late-Onset Bipolar Disorder: A Case Report of New-Onset Mania at Age 65

**DOI:** 10.1192/j.eurpsy.2026.11586

**Published:** 2026-07-31

**Authors:** M. Bouchendira, H. Nefzi, H. Ghabi, L. S. Chaibi, R. Kammoun, M. El Karoui, F. Ellouze

**Affiliations:** 1 Emergencies and consultations; 2 Ibn Jazzar “G”, RAZI hospital, Manouba, Tunisia

## Abstract

**Introduction:**

Bipolar disorder typically has its onset before age 40. Late-onset cases—defined as first manic episodes after age 50—are rare, representing fewer than 10% of new diagnoses after 50 and less than 5% after 60. In parallel to considerable progress in understanding and treatment of bipolarity, and despite growing interest in old age psychiatry, Late-onset bipolar disorder has remained relatively understudied, probably because of its complexity.

**Objectives:**

To report a clinical case of new-onset bipolar disorder in a 65-year-old man and discuss diagnostic, therapeutic, and pathophysiological considerations specific to late-onset presentations.

**Methods:**

We report a clinical case based on medical records from the Department G of Psychiatry at RAZI Hospital.

**Results:**

A 65-year-old man with no prior psychiatric history presented with recurrent manic episodes. Investigations, including neuropsychological testing, laboratory studies, and neuroimaging, excluded organic causes, including dementias and metabolic or structural brain pathology.

He was initially treated with sodium valproate but developed severe refractory thrombocytopenia, preventing continued use. Carbamazepine was also contraindicated. Risperidone was initiated, with partial improvement at 4 mg/day, and clinical stabilization achieved only at 6 mg/day, highlighting the challenges of managing bipolar I disorder in older adults.

Late-onset bipolar disorder is likely a heterogeneous entity. Early- and late-onset cases share clinical symptoms, but different vulnerabilities and etiologies may exist. Understanding the aetiopathogenesis, including the influence of age-related changes is critical for management and prognosis.

**Conclusions:**

Late-onset bipolar disorder, while rare, should be considered once secondary causes are excluded. Management in older adults is challenging due to comorbidities, treatment intolerance, and variable response, necessitating individualized pharmacological strategies, careful titration, and close monitoring. Further studies are required to clarify pathophysiology and optimize treatment.

**Disclosure of Interest:**

None Declared

